# Silver Impairs Neurodevelopment: Studies in PC12 Cells

**DOI:** 10.1289/ehp.0901149

**Published:** 2009-08-31

**Authors:** Christina M. Powers, Nicola Wrench, Ian T. Ryde, Amanda M. Smith, Frederic J. Seidler, Theodore A. Slotkin

**Affiliations:** 1 Department of Pharmacology and Cancer Biology, Duke University Medical Center, Durham, North Carolina, USA;; 2 Nicholas School of the Environment and Earth Sciences, Duke University, Durham, North Carolina, USA

**Keywords:** acetylcholine, developmental neurotoxicity, dopamine, in vitro, metal neurotoxicity, PC12 cells, silver

## Abstract

**Background:**

Exposure to silver is increasing because of silver nanoparticles in consumer products.

**Objectives and methods:**

Many biological effects of silver entail actions of Ag^+^ (monovalent silver ions), so we used neuronotypic PC12 cells to evaluate the potential for silver to act as a developmental neurotoxicant, using chlorpyrifos (CPF), a pesticide known to evoke developmental neurotoxicity, as a positive control for comparison.

**Results:**

In undifferentiated cells, a 1-hr exposure to 10 μM Ag^+^ inhibited DNA synthesis more potently than did 50 μM CPF; it also impaired protein synthesis but to a lesser extent than its effect on DNA synthesis, indicating a preferential effect on cell replication. Longer exposures led to oxidative stress, loss of viability, and reduced numbers of cells. With the onset of cell differentiation, exposure to 10 μM Ag^+^ evoked even greater inhibition of DNA synthesis and more oxidative stress, selectively impaired neurite formation without suppressing overall cell growth, and preferentially suppressed development into the acetylcholine phenotype in favor of the dopamine phenotype. Lowering the exposure to 1 μM Ag^+^ reduced the net effect on undifferentiated cells. However, in differentiating cells, the lower concentration produced an entirely different pattern, enhancing cell numbers by suppressing ongoing cell death and impairing differentiation in parallel for both neurotransmitter phenotypes.

**Conclusions:**

Our results show that silver has the potential to evoke developmental neurotoxicity even more potently than known neurotoxicants, such as CPF, and that the spectrum of effects is likely to be substantially different at lower exposures that do not show signs of outright toxicity.

Each year, thousands of new chemicals are released into the environment, a substantial proportion of which are likely to elicit developmental neurotoxicity (Boyes 2001), thus contributing to the “silent pandemic” of neurodevelopmental disorders (Grandjean and Landrigan 2006). Recently, there has been an explosive increase in the use of silver in medical and consumer products, largely in the form of silver nanoparticles, leading to a corresponding increase in human and ecosystem exposures (Chen and Schluesener 2008). The relatively large surface-to-volume ratio of nanosilver increases the likelihood of oxidation on the particle surface, with consequent release of monovalent silver ions (Ag^+^), the active form that is responsible for the desired antimicrobial effect (Damm et al. 2008; Evanoff and Chumanov 2005). Although silver neurotoxicity in adults occurs only at very high exposures (Lansdown 2007), a number of features may make it more toxic during development. First, silver penetrates the placental barrier and accumulates in the fetus, achieving tissue concentrations of 20 ppb (0.2 μM), much higher than those in the mother, with persistence through the first postnatal year (Lyon et al. 2002). Second, the antimicrobial activity of Ag^+^ relies on its abilities to inhibit DNA synthesis, degrade protein function, and evoke oxidative stress (Feng et al. 2000; Le Pape et al. 2004), the same targets shared by many developmental neurotoxicants (Boyes 2001; Grandjean and Landrigan 2006; Slotkin 2005; Slotkin et al. 2007b). Indeed, the developing brain may provide an environment especially conducive to silver toxicity because of its higher oxygen consumption and unique membrane lipid composition, increased metabolic demand associated with growth, lower reserves of protective enzymes and antioxidants, and low concentrations of glia, which in the adult brain protect neurons by scavenging reactive oxidative species (Gupta 2004; Tanaka et al. 1999). This vulnerability, when combined with the developing brain’s need for carefully coordinated spatiotemporal assembly of complex structures (Bondy and Campbell 2005; Grandjean and Landrigan 2006), thus enhances the likelihood that disruption of cell replication and/or differentiation by Ag^+^ may lead to adverse neurobehavioral consequences.

Few studies have examined the developmental neurotoxicity of silver. In adult rodents, acute exposure to high levels of silver failed to elicit oxidative stress in the brain (Rungby 1987), but chronic exposures produced signs of neuronal damage and consequent behavioral changes (Rungby 1990; Rungby and Danscher 1984); comparable studies of developmental exposures have not been done. As expected from findings in human fetal tissues (Lyon et al. 2002), silver administration to pregnant rats leads to accumulation in the brain of the offspring (Rungby and Danscher 1983); direct administration of silver salts to neonatal rats results in damage to particularly vulnerable areas of the brain, such as the hippocampus (Rungby et al. 1987), but it is not clear whether this damage represents direct effects of silver on neurodevelopment or secondary effects reflecting systemic toxicity. In the present study, we used PC12 cells, a well-defined neurodevelopmental model (Teng and Greene 1994) that readily recapitulates key events in neuronal cell development and thus has served as a model system in numerous studies evaluating exposures to neurotoxicants. In contrast to primary neuronal cultures, which do not undergo mitosis, PC12 cells provide a homogeneous population that continues to divide until differentiation is triggered by addition of nerve growth factor (NGF). Thus, this model allows direct study of effects on DNA synthesis associated with cell replication, which is often a specific target of neurotoxicants (Slotkin 2005). When NGF is added, the cells gradually exit the cell cycle and differentiate into distinct acetylcholine and dopamine phenotypes (Teng and Greene 1994). In the present study, we examined the effects of Ag^+^ exposure on key aspects of cell replication and neurodifferentiation in the PC12 model, including measurements of DNA and protein synthesis, cell number and growth, cell viability, oxidative stress, neurite extension, and differentiation into the two phenotypes. For comparison, in some determinations we included the organophosphate insecticide chlorpyrifos (CPF) as a positive control compound, because this agent is a known developmental neurotoxicant for which the PC12 model provides ready prediction of effects of *in vivo* exposure (Jameson et al. 2006; Qiao et al. 2001, 2005; Slotkin 2005; Slotkin et al. 2007a, 2007b).

## Materials and Methods

All of the techniques used in this study have been reported previously (Jameson et al. 2006; Qiao et al. 2001, 2003, 2005; Song et al. 1998), so only brief descriptions of procedures will be given here.

### Cell cultures

Experiments were performed on cells that had undergone fewer than five passages. PC12 cells (American Type Culture Collection, 1721-CRL; obtained from the Duke Comprehensive Cancer Center, Durham, NC) were grown in RPMI-1640 medium (Invitrogen, Carlsbad, CA) supplemented with 10% inactivated horse serum (Sigma Chemical Co., St. Louis, MO), 5% fetal bovine serum (Sigma), and 50 μg/mL penicillin/streptomycin (Invitrogen); cells were incubated with 5–7.5% CO_2_ at 37°C. For studies in the undifferentiated state, the medium was changed 24 hr after seeding to include 50 μM CPF (98.8% purity; Chem Service, West Chester, PA) dissolved in dimethyl sulfoxide (DMSO), or varying concentrations of silver nitrate (AgNO_3_; Sigma) in water. As a control for any effect of NO_3_^−^ (nitrate ions), we included controls containing NaNO_3_ (sodium nitrate; Sigma), and similarly, where appropriate, controls included DMSO to match the final concentration (0.1%) achieved in the CPF samples; previous studies showed that this concentration of DMSO has no effect on PC12 cell replication or differentiation (Qiao et al. 2001, 2003; Song et al. 1998).

Vitamin E (α-tocopherol; Sigma) was dissolved in 95% ethanol (0.05% final concentration), and controls for these experiments were treated with 0.05% ethanol vehicle. For studies in differentiating cells, 24 hr after seeding, the medium was changed to include 50 ng/mL of 2.5S murine NGF (Invitrogen); each culture was examined under a microscope to verify the subsequent outgrowth of neurites. The test agents were added concurrently with the start of NGF treatment, and cultures were maintained for up to 6 days, with the indicated agents included with every medium change (48-hr intervals).

We chose the CPF concentration to elicit a robust response for each of the effects to be compared with the actions of Ag^+^ but that was less than the threshold for outright cytotoxicity or loss of viability (Jameson et al. 2006; Qiao et al. 2001, 2003, 2005; Slotkin et al. 2007b; Song et al. 1998). Vitamin E was similarly evaluated at concentrations (10 and 30 μM) established for demonstrable prevention of oxidative stress in earlier work (Qiao et al. 2005); ascorbic acid was tested at 10 μM after preliminary studies showed cytotoxicity at 1 mM.

### DNA and protein

Cells were harvested and washed, and the DNA and protein fractions were isolated and analyzed as described previously (Slotkin et al. 2007b; Song et al. 1998). Because neuronal cells contain only a single nucleus, measuring the DNA content in each dish provides a measure of cell number. We measured DNA synthesis by assessment of [^3^H]thymidine incorporation into the DNA fraction (Slotkin et al. 2007b; Song et al. 1998), and similarly, we assessed protein synthesis by incorporation of [^3^H]leucine. To initiate the measurement, the medium was changed to include 1 μCi/mL of either [^3^H]thymidine (specific activity, 2 Ci/mmol; PerkinElmer Life and Analytical Sciences, Boston, MA) or [^3^H]leucine (specific activity, 60 Ci/mmol; PerkinElmer) and the corresponding test agents. One hour later, the medium was aspirated, cells harvested, and the DNA and protein fractions isolated and quantified for radiolabel and for total DNA.

### Oxidative stress

We assessed oxidative stress through measuring the formation of lipid peroxides by reaction of the resultant malondialdehyde (MDA) with thiobarbituric acid (Qiao et al. 2005). To give the MDA concentration per cell, values were calculated relative to the amount of DNA. In experiments testing whether reduction of oxidative stress ameliorated cell loss due to Ag^+^ exposure, we added either vitamin E or sodium ascorbate (Sigma) concurrently with the test agents, as previously established for demonstrable prevention of oxidative stress (Qiao et al. 2005).

### Viability

To assess cell viability, the cell culture medium was changed to include trypan blue (Sigma; 1 vol per 2.5 vol medium), and cells were examined by a blinded observer for staining with a Zeiss Axio Observer (Carl Zeiss MicroImaging, Thornwood, NY) at 100× magnification, counting an average of 400 cells per field in two different fields per culture.

### Enzyme activities

We conducted choline acetyltransferase (ChAT) and tyrosine hydroxylase (TH) assays following published techniques (Lau et al. 1988) using substrates of [^14^C]acetyl coenzyme A (specific activity, 60 mCi/mmol; PerkinElmer) and [^14^C]tyrosine (specific activity, 438 mCi/mmol; PerkinElmer), respectively. Activities were calculated relative to the DNA content.

### Data analysis

All studies were performed on 8–16 separate cultures for each measure and treatment, using two to four separate batches of cells. Results are presented as means and SEs, with treatment comparisons carried out by analysis of variance (ANOVA) followed by Fisher’s protected least significant difference test for post hoc comparisons of individual treatments; data were log-transformed whenever the variance was heterogeneous. In the initial test, we evaluated two ANOVA factors (treatment and cell batch) and found that the results did not vary among the different batches of cells; therefore, results across the different batches were normalized and combined for presentation. Significance was assumed at *p* < 0.05.

## Results

### Undifferentiated PC12 cells

**Figure 1:**
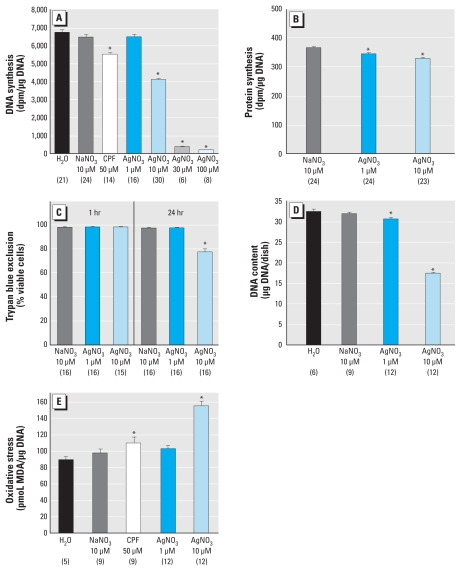
Effects of AgNO_3_ on undifferentiated PC12 cells. DNA synthesis (*A*) and protein synthesis (*B*) after 1 hr exposure (ANOVA for *A* and *B*: treatment, *p* < 0.0001). (*C*) Trypan blue exclusion after 1 hr and 24 hr exposure (ANOVA: treatment, *p* < 0.0001; treatment × time, *p* < 0.0001). DNA content (*D*) and oxidative stress (*E*) after 24 hr exposure (ANOVA for *D* and *E*: treatment, *p* < 0.0001). Data represent mean ± SE obtained from the number of determinations shown in parentheses. In *A* and *E*, 50 μM CPF was included as a positive test compound for comparison with AgNO_3_. *p < 0.05 compared with the corresponding control group.

Even a 1-hr exposure of undifferentiated cells to Ag^+^ reduced DNA synthesis, with a threshold concentration between 1 and 10 μM (Figure 1A); equivalent concentrations of NaNO_3_ did not produce any decrement, indicating that the effects were related to Ag^+^ rather than NO_3_^−^. Further increases to 30 or 100 μM resulted in near-total mitotic arrest. To determine whether this inhibition was specific to cell replication compared with general cytotoxicity or global effects on macromolecular synthesis, we evaluated protein synthesis under the same conditions (Figure 1B). Although Ag^+^ evoked a statistically significant decrement in protein synthesis, the magnitude of effect was substantially smaller than for DNA synthesis; notably, however, we found a significant effect even at 1 μM Ag^+^, which pointed to the need to examine longer-term effects at this lower concentration. The effects on macromolecule synthesis were not secondary to loss of cell viability, because we found no decrease in the ability of the cells to exclude trypan blue at the 1 hr time point (Figure 1C); however, by 24 hr of exposure, we observed a significant decrease in viability at 10 μM Ag^+^ but not at the lower concentration. The combined effect of antimitotic actions and decreased viability produced a significant decrement in DNA content after 24 hr exposure to 10 μM Ag^+^ (Figure 1D); however, we found a significant, albeit slight, loss even at the lower Ag^+^ concentration. Undifferentiated cells also displayed oxidative stress in response to a 24 hr exposure to 10 μM Ag^+^ (Figure 1E). Again, we did not observe this effect with equivalent NaNO_3_ concentrations, and it was greater in magnitude than that seen with 50 μM CPF. The lower Ag^+^ concentration evoked a smaller elevation in MDA just above the threshold for statistical significance (*p* < 0.08).

Because Ag^+^ salts have limited solubility, we performed additional experiments using light scattering to assess whether Ag^+^ was forming particulates. We examined seven wavelengths spanning the visible spectrum. In water, we found no increase in optical density at 1 μM AgNO_3_, but we did see small increments of 7–9% at 10 or 100 μM (data not shown). However, when AgNO_3_ was added to the culture medium, we observed no significant increases until the concentration was raised to 100 μM (8% increase), indicating that factors in the medium complexed the Ag^+^ sufficiently to keep it in solution. To confirm complexation, we examined DNA synthesis during a 1-hr incubation in the presence and absence of serum proteins; this period is short enough for viability to be maintained without serum (Qiao et al. 2001). In the absence of serum, 10 μM Ag^+^ was far more effective in inhibiting DNA synthesis than when serum was present: with serum, 2,273 ± 114 disintegrations per minute (dpm)/μg for control and 1,977 ± 156 dpm/μg for 10 μM Ag^+^; without serum, 3,295 ± 145 dpm/μg and 274 ± 82 dpm/μg, respectively (*n* = 4).

### Differentiating PC12 cells

**Figure 2:**
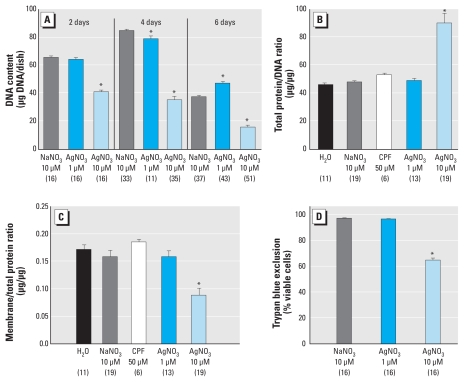
Effects of AgNO_3_ on differentiating PC12 cells. (*A*) DNA content after 2, 4, or 6 days (ANOVA: treatment, *p* < 0.0001; treatment × time, *p* < 0.0001). Total protein/DNA ratio (*B*) and membrane/total protein ratio (*C*) 6 days after exposure (ANOVA for *B* and *C*: treatment, *p* < 0.0001). (*D*) Trypan blue exclusion 4 days after exposure (ANOVA: treatment, *p* < 0.0001). Data represent mean ± SE obtained from the number of determinations shown in parentheses. In *B* and *C*, 50 μM CPF was included as a positive test compound for comparison with AgNO_3_. Absolute values for DNA content cannot be compared across the different time points because cells were plated at different densities to achieve equivalent confluence at the time of sampling. *p < 0.05 compared with the corresponding control group.

Continuous exposure of differentiating PC12 cells to 10 μM Ag^+^ resulted in a progressive deficit in cell numbers, as delineated by the effects on DNA content (Figure 2A). We verified that the DNA measurements reflected the actual cell number by counting cells at the 4-day time point (*n* = 16). Ag^+^ (10 μM) produced a 60% decrement in cell numbers, the same percentage as for DNA content: 535 ± 15 cells/field for the NaNO_3_ control, 501 ± 12 for 1 μM Ag^+^, and 209 ± 10 for 10 μM Ag^+^ (*p* < 0.0001). Notably, the lower Ag^+^ concentration elicited a biphasic effect, with subnormal values after 4 days of exposure and elevated values after 6 days. The dichotomy between the two doses was also reflected in the impact on parameters of cell growth and viability. The high Ag^+^ concentration, but not the low concentration, evoked an increase in the total protein/DNA ratio (Figure 2B), a decrease in the membrane/total protein ratio (Figure 2C), and a loss of viability (Figure 2D).

**Figure 3:**
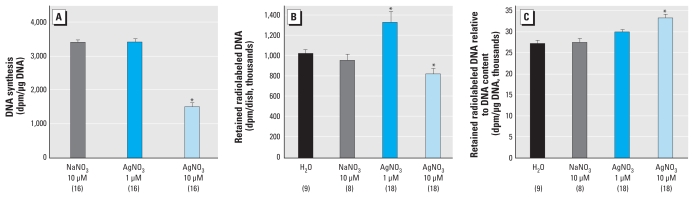
DNA synthesis and retention of radiolabeled DNA in differentiating PC12 cells. In (*A*) cells were exposed to test agents simultaneously with NGF for 2 days, and then [^3^H]thymidine incorporation into DNA was measured for a 1-hr period at the end of exposure. In (*B*), undifferentiated cells were radiolabeled with [^3^H]thymidine; cells were then exposed simultaneously to test agents with NGF for 6 days, and the radiolabel retained in the DNA fraction was determined. (*C*) Retained radiolabel after 6 days relative to the DNA content. Data represent mean ± SE obtained from the number of determinations shown in parentheses. For *A*–*C*, ANOVA: treatment, *p* < 0.0001. *p < 0.05 compared with the corresponding control group.

To explore the reason for the unexpected increase in DNA content evoked by exposure to 1 μM Ag^+^ compared with the decrease seen at 10 μM, we performed several additional experiments. First, we determined whether Ag^+^ delayed the transition from cell replication to cell differentiation, which would result in accumulation of excess cells despite the presence of NGF. We exposed cells to test substances simultaneously with NGF for 2 days and then determined the residual rate of DNA synthesis at the end of that period (Figure 3A). A delay in the transition should result in an elevated rate of DNA synthesis relative to differentiating control cells. As expected, the DNA synthesis rate in NaNO_3_-exposed control cells was much lower after the 2-day exposure to NGF than in undifferentiated cells (compare control values in Figure 3A with those in Figure 1A). The high concentration of Ag^+^ again suppressed DNA synthesis, but we saw no effect of exposure to 1 μM Ag^+^. The second set of studies explored whether excess cell accumulation might reflect a decrease in the rate of spontaneous cell loss that accompanies PC12 cell differentiation (Ekshyyan and Aw 2005). We prelabeled the DNA by exposing undifferentiated, replicating PC12 cells to [^3^H]thymidine for 24 hr, after which the medium was changed to include NGF and the test substances, without further addition of radiolabel. At the end of 6 days, we assessed how much of the originally labeled DNA remained. The high concentration of Ag^+^ evoked a significant decrement in retained radiolabel, whereas the low concentration produced a substantial increase (Figure 3B). Because we also found changes in DNA content reflecting alterations in the number of cells (Figure 2A), we further examined the retention of radiolabel relative to cell number (Figure 3C). For this parameter, 1 μM Ag^+^ evoked a small increment at the margin of statistical significance (*p* < 0.06), whereas 10 μM Ag^+^ evoked a more substantial, significant increase; this means that, for both treatments, cells formed before we added Ag^+^ survived longer than those generated in the presence of Ag^+^.

**Figure 4:**
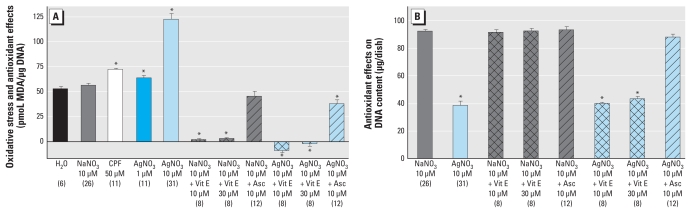
Oxidative stress and antioxidant effects in differentiating PC12 cells after 4-day exposure. MDA levels (*A*) and DNA content (*B*). Data represent mean ± SE obtained from the number of determinations shown in parentheses. Abbreviations: Vit E, vitamin E; Asc, ascorbate. In *A*, 50 μM CPF was included as a positive test compound for comparison with AgNO_3_. For *A* and *B*, ANOVA: treatment, *p* < 0.0001 *p < 0.05 compared with the corresponding control group.

Given our observation of oxidative stress resulting from Ag^+^ exposure of undifferentiated cells, we performed comparable studies with exposure during differentiation, focusing on an intermediate time point (4 days) so as to examine effects before the terminal effects on cell number. Exposure to either 1 or 10 μM Ag^+^ evoked significant increases in lipid peroxidation, and again, the effect of 10 μM Ag^+^ was greater than that achieved with 50 μM CPF (Figure 4A). To explore the role of oxidative stress in the cell loss evoked by the higher Ag^+^ concentration, we then assessed the impact of antioxidant treatments on lipid peroxidation and cell number. Treatment with 10 μM or 30 μM vitamin E alone reduced MDA levels to near zero, whereas 10 μM ascorbate caused only a small, nonsignificant reduction. However, all three treatments prevented the lipid peroxidation evoked by 10 μM Ag^+^, reducing the MDA level to zero (vitamin E) or to lower values than in controls (ascorbate). In contrast, the impact of the two antioxidants on DNA content was quite different (Figure 4B). By themselves, neither vitamin E nor ascorbate had any effect. Despite the reversal of lipid peroxidation, vitamin E did not provide protection from the cell loss evoked by 10 μM Ag^+^, whereas ascorbate was completely protective. We carried out one additional experiment to determine whether cell loss from 10 μM exposure was due to its interference with copper uptake via competitive inhibition of Cu transport (Bertinato et al. 2008). However, addition of equimolar concentrations of Cu^2+^ failed to ameliorate cell loss due to 10 μM Ag^+^ exposure (data not shown).

**Figure 5:**
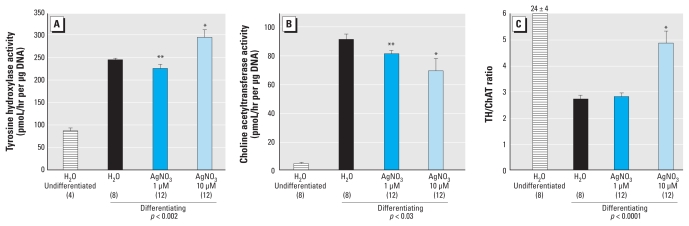
Effects of AgNO_3_ on differentiation of PC12 cells into dopamine and acetylcholine phenotypes, assessed by measuring TH and ChAT, respectively. (*A*) TH activity. (*B*) ChAT activity. (*C*) TH/ChAT ratio. Data represent mean ± SE obtained from the number of determinations shown in parentheses. ANOVA for effects in the differentiating cells appears at the bottom of each panel. *p < 0.05 compared with the corresponding control group. **Significant main effect of AgNO_3_ exposure that was equivalent for TH and ChAT, as assessed by a two-factor ANOVA incorporating treatment and measure (TH and ChAT as a repeated measure because both enzymes were assayed in the same sample).

Finally, we assessed the effects of Ag^+^ exposure on differentiation of PC12 cells into neurotransmitter phenotypes. In control cells exposed to NGF for 6 days, TH activity was much higher than in undifferentiated cells, reflecting formation of the dopamine phenotype (Figure 5A). Coexposure to 1 μM Ag^+^ evoked a small reduction in TH, whereas the higher concentration elicited a significant increase. For ChAT, NGF exposure in control cells evoked an even larger proportional increase compared with the undifferentiated state, reflecting formation of the acetylcholine phenotype (Figure 5B); in this case, 1 and 10 μM Ag^+^ produced effects in the same direction (decreased ChAT). Although the effects of 1 μM Ag^+^ on TH and ChAT did not achieve statistical significance individually, a higher-order statistical analysis of the results—considering factors of both treatment and measure (TH and ChAT as a repeated measure, because both enzymes were assayed in the same sample)—indicated a significant main treatment effect, reflecting a significant overall decrement across both phenotypes. The differences in outcome between 1 μM and 10 μM Ag^+^ were more obvious when considering the TH/ChAT ratio as a measure of phenotypic preference (Figure 5C). The lower Ag^+^ concentration had no effect on the ratio, reflecting the equivalent decrements seen for both phenotypes, whereas the higher concentration evoked a significant increase, indicating a switch away from the acetylcholine phenotype and toward the dopamine phenotype. Exposure to NaNO_3_ had no effect (data not shown).

## Discussion

Our results show that Ag^+^ can potentially elicit developmental neurotoxicity at concentrations orders of magnitude lower than required to elicit the same effects with CPF, a known neurotoxicant. Notably, however, the outcomes and underlying mechanisms were substantially different at concentrations greater than and less than the threshold for direct cytotoxicity, and also were influenced by whether cells were undergoing replication or differentiation. Accordingly, the effects of Ag^+^ exposure on neuronal development are highly stage dependent and display nonmonotonic concentration–effect relationships.

Exposure of undifferentiated PC12 cells to 10 μM Ag^+^ produced an immediate decline in DNA synthesis, progressing to near-total inhibition at higher concentrations. The effect was much greater than that exerted toward protein synthesis and preceded the loss of cell viability, thus indicating selective antimitotic activity. This conclusion was reinforced by measurements made after 24 hr exposure, indicating substantially larger deficits (50%) in cell numbers than could be accounted for solely by reduced cell viability (20%). The effects were accompanied by a substantial degree of oxidative stress, which likely provides a major contribution to both outcomes. However, the differences in time of onset and degree of effect indicate that Ag^+^ directly inhibits mitotic activity. Indeed, in bacteria, Ag^+^ exposure produces DNA condensation, likely as a reaction to protect the genetic information from interaction with the toxicant (Feng et al. 2000); however, in the context of brain development, condensing DNA would impair cell replication precisely during the critical period in which each neuronal population is being generated (Bondy and Campbell 2005). Accordingly, if the same events occur in mammalian neurodevelopment, we would expect to see widespread neuronal deficits corresponding to the regions undergoing neurogenesis during the exposure period.

We observed even greater cell loss in cells exposed to 10 μM Ag^+^ during differentiation. In part, this represented an accelerated decline in mitotic activity, likely involving mechanisms similar to those seen in the undifferentiated state. Indeed, 10 μM Ag^+^ elicited oxidative stress and an even larger decline in viability than that seen in undifferentiated cells. Nevertheless, we again observed a number of selective effects independent of the more general cytotoxic actions. First, the protein/DNA ratio was increased, reflecting greater effects on cell number than on cell growth; indeed, the rise in the protein/DNA ratio is consistent with enlargement of the remaining cells (Slotkin et al. 2007b; Song et al. 1998), again consistent with selective targeting of cells undergoing mitosis rather than general growth impairment. Prelabeling cells with [^3^H]thymidine before commencing Ag^+^ exposure revealed a preferential loss of cells that formed in the presence of the toxicant, as reflected by a greater decrease in total cell number than in the prelabeled cells (i.e., an increase in the [^3^H]thymidine/DNA ratio); if Ag^+^ were instead simply acting through general cytotoxicity, then loss of radiolabel would have been equivalent to cell loss. The greater vulnerability of nascent cells to Ag^+^ could reflect either a greater impact on cell function or, alternatively, greater uptake of the toxicant, a distinction that needs to be addressed in future studies. However, regardless of the underlying mechanism, the heightened susceptibility of newly formed cells reinforces the concept that there are critical neurodevelopmental stages especially sensitive to Ag^+^.

In support of that conclusion, we then confirmed a specific effect of Ag^+^ on neurodifferentiation itself, in terms of indices of neurite formation and development into neurotransmitter phenotypes. Exposure to 10 μM Ag^+^ reduced the membrane/total protein ratio, reflecting impaired formation of neurites (Slotkin et al. 2007b; Song et al. 1998), and suppressed expression of the acetylcholine phenotype, as reflected by a significant decrease in ChAT. Interestingly, however, the same exposure enhanced differentiation into the dopamine phenotype (elevated TH activity); the combined effects produced an even larger increase in the TH/ChAT ratio, indicating diversion of neurodifferentiation away from the acetylcholine phenotype and toward the dopamine phenotype. If similar effects occur in the developing brain, then Ag^+^ would likely impair axonogenesis/synaptogenesis, as well as produce “miswiring,” where presynaptic neurons would contain a neurotransmitter inappropriately matched to the postsynaptic receptors on their target cells.

We also examined the relationship between oxidative stress and cytotoxicity by evaluating whether antioxidants could prevent the cell loss caused by exposure of differentiating cells to 10 μM Ag^+^. Although vitamin E completely suppressed lipid peroxidation, it failed to prevent the loss of cells, despite the fact that, in earlier work, we found that the same treatment provided partial defense against oxidative damage caused by CPF (Slotkin et al. 2007a). However, ascorbate provided complete protection against cell loss, even though it had a lesser effect on lipid peroxides. There are a number of possible explanations for the dichotomy in the effects of vitamin E and ascorbate, each of which points to potentially important mechanisms. First, ascorbate is a cytosolic reducing agent, whereas vitamin E, because of its high partition coefficient, concentrates in membranes. Accordingly, ascorbate might quench the activity of Ag^+^ in the cell by reducing the ion to Ag^0^, which is more readily confined to intracellular organelles than the charged species. By itself, the redox potential for ascorbate is insufficient to drive the reaction Ag^+^ → Ag^0^ at the equimolar concentrations that we included in the culture medium, but the intracellular level of ascorbate is likely to be much greater than that of Ag^+^; alternatively, higher ascorbate concentrations may foster enzymatic reduction of the metal. A second possibility is that, because vitamin E acts downstream from the production of reactive oxygen species to minimize the end point of lipid peroxidation within the cell membrane, our results could point to targets other than membrane lipids that might be more sensitive to the oxidative stress evoked by Ag^+^. Third, ascorbate might interfere with the entry of Ag^+^ into the cell, which likely requires a transporter (Bertinato et al. 2008). We intend to explore these possibilities in future work, but in any case, our findings confirm that oxidative stress per se, although contributing to the effects of Ag^+^ on neurodevelopment, cannot solely account for all the observations; this is further reinforced by the dichotomy between effects of Ag^+^ at 10 μM compared with 1 μM.

Reducing the Ag^+^ concentration to 1 μM had the expected, lowered effect on undifferentiated PC12 cells. Inhibition of DNA synthesis was reduced to the point of nonsignificance (although protein synthesis remained slightly affected), and there was no detectable loss of viability. Oxidative stress was lowered to the threshold of significance, and cell loss after 24 hr of exposure was quite minor, albeit still detectable. We were therefore surprised to find a substantially different spectrum of actions for the effects of 1 μM Ag^+^ in differentiating cells compared with effects seen at the higher concentration. First, the low concentration elicited an increase in cell numbers rather than a decrease, with the effect emerging between 4 days and 6 days of exposure. This did not reflect a delay in the transition from replication to differentiation because the treatment did not impair the ability of NGF to elicit a decline in DNA synthesis. However, when we prelabeled the DNA with [^3^H]thymidine before commencing coexposure to 1 μM Ag^+^ and NGF, we found an increased retention of radiolabel after 6 days, pointing to a sparing of ongoing apoptotic cell loss (Ekshyyan and Aw 2005) as the likely mechanism underlying excess cell accumulation. Because apoptotic cell loss is an essential feature of normal brain assembly (Kuida et al. 1996), if similar effects occur *in vivo*, then we would expect to see substantial alterations in architectural modeling of brain regions in which active apoptosis is occurring. Also unlike the higher concentration, 1 μM Ag^+^ did not affect parameters of cell growth or neurite formation, but it did affect neurodifferentiation into acetylcholine and dopamine phenotypes. Again, however, the pattern differed substantially from those seen at 10 μM; at 1 μM, we observed impairment of both TH and ChAT to approximately the same extent. We would therefore expect to see deficits in the emergence of both phenotypes but without the preferential switch to the dopamine phenotype that we saw at 10 μM Ag^+^. With *in vivo* exposures, we would also predict correspondingly different patterns of neurobehavioral outcomes between exposures greater than and less than the threshold for outright toxicity.

The dichotomies in the effects of 1 μM versus 10 μM suggest that multiple mechanisms participate in the developmental neurotoxicity of Ag^+^. Although these remain to be elaborated, one possible contributor is the different degrees of oxidative stress achieved at the two concentrations. In differentiating cells, 1 μM Ag^+^ elicited a significant increase in lipid peroxidation, but the effect at the higher concentration was more than six times greater. An increase in intracellular oxidative status is normally an important trophic signal in cell differentiation (Katoh et al. 1997), and thus, a small degree of oxidative stress is likely to produce very different outcomes from those seen at higher oxidative levels that produce cell damage and cytotoxicity (Kamata and Hirata 1999). Certainly, the distinction between trophic and toxic oxidative end points could contribute to the observation of decreased cell death at 1 μM Ag^+^ but increased cell loss at 10 μM. Nevertheless, as pointed out above, oxidative stress alone cannot account for all the observations, and future work will need to address the additional contributory mechanisms. In any case, it is clear that lower Ag^+^ exposures do not simply produce a smaller net impact on neurodevelopment, but rather display a different spectrum of effects.

The limited solubility of Ag^+^ salts in aqueous solutions may provide a second contributor to nonmonotonic dose–effect relationships. The solubility product for AgCl is very low, and as found here, formation of insoluble particulates occurs in water beginning at 10 μM AgNO_3_. Indeed, the formation of microparticles or individual molecules of electroneutral AgCl is probably important for its penetration through membrane barriers, including the blood–brain barrier, the placental barrier, and the cell membrane, all of which exclude charged moieties. In addition, Ag^+^ is a substrate for the Cu^+^ transporter (Bertinato et al. 2008), and it is also conceivable that microparticles may form that are of the correct size for endocytotic uptake into the cell. In any case, we found that complexation with serum proteins in the culture medium maintained Ag^+^ in solution at higher concentrations and thus also protected against cell damage. This may be quite relevant to developmental neurotoxicity, because the fetus and neonate possess lower levels of serum proteins (Yaffe and Stern 1976) and thus would be likely to show toxicity at lower total Ag^+^ concentrations.

Our use of an *in vitro* system to evaluate the developmental neurotoxicity of Ag^+^ has both advantages and disadvantages compared with *in vivo* models. Obviously, *in vitro* models enable us to determine the direct impact of Ag^+^ on neuronal cell replication and differentiation because they lack many of the confounding variables that operate in mammalian systems, such as indirect effects on maternal nutrition, endocrine status, and behavior. Additionally, because PC12 cell differentiation is triggered by treatment with NGF, we can examine effects at specific neurodevelopmental stages ranging from initial cell replication, to the transition to differentiation, and then to the expression of specific neurotransmitter phenotypes. In contrast, primary neurons do not divide in culture, do not show a uniform pattern of differentiation, and have a wide variety of phenotypes. At the same time, the *in vitro* model lacks the ability to assess pharmacokinetic factors that influence fetal exposure, and it underestimates the sensitivity to toxicants. The latter reflects a combination of several factors. As a transformed cell line, PC12 cells are inherently more resistant to drugs and chemicals and typically require much higher concentrations to display effects that are seen at lower concentrations in intact neurons or in the developing brain. Second, exposures in culture occupy a time frame of hours to days, whereas exposures *in vivo* can involve exposure over the entire course of brain development; again, this reduces the apparent sensitivity of *in vitro* models. Third, the cell culture model lacks many of the important aspects that define architectural assembly of the brain, notably the cell-to-cell interactions and larger-scale events that simply cannot be modeled *in vitro*. Nevertheless, all these features combine to make *in vitro* models generally less sensitive to Ag^+^, so comparable effects *in vivo* are likely to be seen at much lower concentrations than those used here. Finally, the very confounders that are eliminated with *in vitro* models are of considerable importance in risk assessment, which has to take into account maternal endocrine status and other indirect sources of toxicity (Hartung and Daston 2009).

Our results provide some of the first evidence that Ag^+^ acts directly as a developmental neurotoxicant. At high concentrations, Ag^+^ inhibits cell replication and enhances cell death, leading to a loss of cells, as well as retarding neurite formation and switching neurodifferentiation away from the acetylcholine phenotype and toward the dopamine phenotype. At low concentrations, Ag^+^ retards normal apoptotic cell death and impairs neurodifferentiation into both phenotypes. The *in vitro* model thus points to the neurodevelopmental processes that are targeted at each concentration, which in turn can guide *in vivo* investigations toward the examination of specific neuronal populations, brain regions, and the probable critical periods of heightened vulnerability. Notably, the effects seen here involved concentrations less than an order of magnitude greater than those found historically in human fetal tissues; in light of the explosive increase in the use of silver in consumer products, exposures can be expected to increase substantially over the next few years (Chen and Schluesener 2008). Given that the PC12 model has numerous factors that reduce its sensitivity to toxicant exposure, and in light of standard regulatory requirements for orders-of-magnitude margins of safety, our findings point to the likelihood that *in vivo* exposures to Ag^+^ and silver nanoparticles will likewise lead to developmental neurotoxicity.
